# Digitally supported participation in the nexus between public health and urban planning

**DOI:** 10.1007/s00103-024-03838-0

**Published:** 2024-02-08

**Authors:** Rehana Shrestha, Pia Hasselder, Gabriele Bolte

**Affiliations:** 1 Institute of Public Health and Nursing Research, Department of Social Epidemiology, University of Bremen, Bremen, Germany; 2 Leibniz Science Campus Digital Public Health Bremen, Bremen, Germany

**Keywords:** Digital participation, Healthy urban planning, Interaction, Empowerment, Environmental justice, Digital unterstützte Partizipation, Gesundheitsfördernde Stadtentwicklung, Interaktion, Empowerment, Umweltgerechtigkeit

## Abstract

The nexus between urban planning and public health acknowledges the importance of creating cities that contribute to their residents’ physical, mental, and social well-being. The Healthy Cities movement underlines that community participation and intersectoral work are important to create sustainable, equitable, and healthy cities.

Several theoretical and practical participatory approaches form the foundation for participation in public health and urban planning. Growing digitalization has significantly transformed how participation is conducted in various fields. Digital technologies not only play a large role in daily life, but they have opened more opportunities for individuals to interact, share, and collaborate in the planning and design of cities.

This article explores how digital technologies enable participation among residents and stakeholders in order to support the health-oriented planning of cities and neighborhoods. From the selective case studies presented in the paper, it can be ascertained that digital technologies can support various forms of participation by enabling different levels of engagement as well as both one-way and two-way interactions. Some forms of engagement can be supported entirely within digital platforms. However, in the case of higher engagement, which requires deeper insights into the problems and the codevelopment of solutions, other nondigital formats and traditional methods such as follow-up workshops and focus group discussions are necessary to complement the digital form of participation.

## Abstract (de)

Die Verknüpfung von Stadtplanung und Public Health verdeutlicht, wie wichtig es ist, Städte zu schaffen, die zum körperlichen, geistigen und sozialen Wohlbefinden ihrer Bewohnenden beitragen. Die Gesunde-Städte-Bewegung hebt hervor, dass die Beteiligung der Bevölkerung und die sektorübergreifende Zusammenarbeit sehr wichtig sind für die Gestaltung nachhaltiger, gerechter und gesunder Städte. Verschiedene theoretische und praktische Ansätze bilden die Grundlage für Partizipation in den Bereichen Public Health und Stadtplanung. Die zunehmende Digitalisierung hat die Art und Weise, wie Partizipation umgesetzt wird, erheblich verändert. Digitale Technologien spielen nicht nur im täglichen Leben eine große Rolle, sondern erweitern auch die Beteiligungsmöglichkeiten von Bewohnenden bei der Planung und Umsetzung stadtplanerischer Maßnahmen. Dieser Artikel untersucht, wie digitale Technologien die Beteiligung von Bewohnenden und Interessenvertretenden ermöglicht, um eine gesundheitsorientierte Stadtplanung zu unterstützen. Ausgewählte Fallstudien zeigen, dass digitale Technologien verschiedene Beteiligungsformen unterstützen können, indem sie unterschiedliche Ebenen des Engagements und ein- oder zweiseitige Interaktionen ermöglichen. Einige Formen des Engagements können vollständig durch digitale Plattformen unterstützt werden. Im Falle eines größeren Engagements, das tiefere Einblicke in die Probleme und die gemeinsame Entwicklung von Lösungen erfordert, sind jedoch andere, nichtdigitale Formate und traditionelle Methoden wie Workshops und Fokusgruppendiskussionen notwendig, um die digitale Form der Beteiligung zu ergänzen.

## Introduction

Public health and urban planning share holistic and integrated approaches to city development. The nexus between public health and urban planning acknowledges the intricate and interconnected relationships between the physical and social environmental aspects of urban areas and the well-being of their residents [1]. The intersection between public health and urban planning underlines the importance of creating cities that are economically efficient and contribute to their residents’ physical, mental, and social well-being. It recognizes that the design and organization of urban spaces can have profound effects on health outcomes, making collaboration between these two fields crucial in order to create livable, sustainable, equitable, and healthy cities. Moreover, this relationship is not unidirectional but bidirectional. This means that urban planning decisions can influence health outcomes, and the health of residents can, in turn, influence the effectiveness and sustainability of urban planning strategies.

In an effort to connect these two fields, the healthy urban planning concept emerged from the historical challenges of industrialized cities, the recognition of environmental injustice and health disparities in urban areas, and a growing understanding of the intricate connections between urban planning and public health [2]. Several entry points for health to engage as an input and outcome have been identified in urban design and planning. For example, health-oriented urban design and planning become relevant in settings such as public spaces, schools, and residential estates. Similarly, initiatives in sectors such as housing, education, and transport can provide entry points for health [3]. Following this recognition, important movements such as the Healthy Cities movement started in 1986 within the European region of the World Health Organization (WHO) in order to translate the WHO’s Health for All strategy into local programs and planning [4].

One of the key principles of healthy urban planning is to ensure community participation and multisectoral work to develop inclusive, sustainable, and environmentally just plans and programs that promote residents’ well-being in cities [5, 6]. Community participation generally involves residents, stakeholders, and community members in decision-making that affects their neighborhoods and lives. It recognizes that active engagement of community members in discussions, planning, and decision-making related to their neighborhoods is essential to gain their valuable insights, local knowledge, and expertise. Ideally, community participation incorporates the opinions of residents and community members when policies, programs, and projects are designed. This fosters a sense of ownership among the residents. It empowers individuals and communities to advocate for their priorities and needs and to collaborate with government and other stakeholders, and it ensures that decisions are informed by the voices of those directly affected [7].

Multisectoral work or intersectoral participation refers to the collaboration between different sectors, disciplines, and practitioners. It recognizes that many challenges, especially those related to public health and urban planning, are multifaceted and require contributions from various areas of expertise. With growing recognition of social determinants of health, researchers have agreed that social, behavioral, and environmental factors play a substantial role in the health outcomes of a population [8]. Thus, a collaboration of practitioners from diverse sectors (e.g., public health, urban and environmental planning, education, housing, transport) becomes crucial. Breaking down silos through information and knowledge sharing helps to achieve a more coordinated and informed approach and to address underlying social determinants of health that span multiple sectors [9].

To conclude, community participation emphasizes the involvement of residents and community members in decision-making processes to ensure that interventions are relevant, inclusive, and supported by the community and is referred to as “community partner.” Intersectoral participation, referred to as “practice partner,” focuses on collaboration across various sectors and their practitioners to develop holistic solutions [10]. Both perspectives are valuable in public health and urban planning and complement each other to create healthy living for all, as highlighted by researchers for health-focused urban planning in Germany [11].

Several theoretical and practical participatory approaches have guided and informed participation in various fields, including health and urban planning. These approaches provide a nuanced understanding of the dynamics of participation and factors that influence successful participation. For example, Arnstein’s ladder of citizen participation [12] is a well-known model that depicts different levels of participation, from nonparticipation to higher levels of participation. The ladder highlights the varying degrees of influence that community members can have on decision-making. In the empowerment theory, participation is viewed as a means to build the capacity of communities to advocate for their needs and influence decision-making [13]. It emphasizes the need to develop knowledge and resources so that individuals and communities can take control of their own lives and environments.

Similarly, approaches such as community-based participatory research (CBPR) and participatory action research have been applied extensively to involve community members in researching their challenges [14]. Such approaches incorporate action-oriented research to empower communities to drive change based on their insights and experiences. In the context of intersectoral participation, approaches such as transdisciplinary collaboration approach and coproduction/cocreation/colearning are being emphasized to promote collaboration between disciplines and sectors in order to encourage diverse experts and stakeholders to work together by combining their knowledge and expertise for collaborative creation of innovative, context-specific solutions [15, 16]. Similarly, to ensure participation in research and practice, several community-engaged methods and tools can be applied throughout the process, for example when identifying problems, developing research questions and objectives, designing and conducting the study, and disseminating findings. These include both qualitative and quantitative methods and are used in diverse settings. Duea et al. [17] provide an overview of participatory methods categorized into five domains: engagement and capacity building (e.g., CBPR charrettes, community advisory boards), exploration and visioning (e.g., concept mapping, world café), visual and narrative (participatory geographic information system [GIS] mapping, photovoice, video voice), mobilization (e.g., citizens’ juries, citizens’ panels, Delphi process), and evaluation (e.g., ripple effects mapping).

Recently, advancements in digitalization have significantly transformed how participation can be conducted in various fields. However, these advancements did not happen in a single period. They can be traced back to development over time, particularly from the late 20th century when personal computers started to emerge, laying the foundation for how people interact and exchange ideas. In the early 1990s, particularly during the popularization of the Internet and the World Wide Web, early online forums and websites opened possibilities for individuals to connect and share information globally. In the 2000s, social media platforms such as Facebook, Twitter, YouTube, and user-generated content such as blogs, wikis, and online collaboration tools enabled communities to engage in real-time discussions, create and share content, and foster online campaigns. In the 2010s, mobile technology, apps, digital platforms, and mapping tools further expanded digital participation. This enabled crowdsourcing data and citizen science projects where people can contribute data on their neighborhood environment to inform research and decision-making. Currently, virtual and augmented reality are emerging as new ways to offer immersive experiences to stakeholders and residents to visualize urban plans and design, help them understand proposed plans, and collect feedback [18]. Similarly, the COVID-19 pandemic has further accelerated the use of digital tools for participation, such as virtual meetings, webinars, and online workshops [19].

It can therefore be ascertained that digitalization has not only penetrated individuals’ daily lives but has revolutionized and democratized community participation in planning and design. Digital participatory methods and approaches have the potential to enable individuals and communities to have a direct voice in decision-making processes that affect their neighborhoods. Increasingly, studies are leveraging digital technologies and online platforms to facilitate the participation of communities and stakeholders in the planning and decision-making processes. Nevertheless, several studies have raised concerns about the digital divide and further exacerbation of existing disparities in participation [20, 21]. Therefore, it is necessary to explore the extent to which digital technologies are enabling participation among communities and stakeholders to inform the health-oriented planning and design of cities and neighborhoods.

This paper provides a conceptual framework for digital participation and describes and discusses selected examples of how digital technologies are being used in a participatory context on the topics related to healthy urban planning.

## From participation to digital participation in healthy urban planning

Promoting participation of communities and collaboration across sectors is at the heart of the WHO European Healthy Cities Network to achieve the objectives of healthy urban planning. Drawing upon the Ottawa Charter for Health Promotion [22], participation in healthy urban planning emphasizes community capacity, empowering individuals and promoting intersectoral cooperation. It underlines the necessity of participation with actions being carried out by and with people, not on or to people. However, although healthy urban planning strategy advocates for high levels of participation, promoting direct influence and control over planning decisions, it also acknowledges that the participation of communities and stakeholders operates at different levels and embraces a range of practices as initially conceptualized by Arnstein’s ladder of participation [12].

Despite being a valuable conceptual framework, the Arnstein ladder has faced criticism for being overly hierarchical [23]. The ladder’s linear progression from nonparticipation to participation simplifies the diverse and multidimensional nature of participation and seems to give the impression that the lower rungs of participation are less important than higher rungs. However, participation in a healthy urban planning context may involve a combination of levels and multiple stakeholders, including residents and practitioners, and does not always follow a linear progression. Participants may move between levels based on the issues at hand, their capacity, and the willingness of the proponents of participatory activity to engage. To address this, researchers advocate for a more flexible framework and adapt the earlier conceptualization of participation typologies depending on context and considering the unique dynamics of each situation, for example the wheel of participation by Davidson [24] or Wright’s participation ladder [25].

For this paper, we adopt the continuum of participation presented by the International Association of Participation (IAP2) [26]. This spectrum describes five levels of participation: *inform, consult, involve, collaborate, empower*. It presents the varying levels of participation as a continuum of influence and suggests that different levels offer varying degrees of opportunities for involvement depending on particular social, economic, and organizational contexts as well as goals, time frames, and resources. Additionally, instead of providing information to the participants, we have interpreted the *inform level* as a “bottom-up information” level (Table 1) suggesting that participants are contributors rather than recipients of information. In this respect, even during the information and consultation activity during which the contributions of residents and practitioners might be limited to receiving, sharing information, or providing feedback on already developed projects, plans, and policies, there is an opportunity for the proponent of participatory activity to learn about community, sectoral concerns, and priorities related to health. When such engagement becomes more profound, such as during involvement, collaboration, and empowerment, residents and practitioners gain more influence on plans and projects. They become active members and enable shared decision-making and cocreation strategies to promote health and well-being.

**Table 1:** Interaction format and participatory format across the continuum of participation

Bottom-up information (data collection)	Consultation (sharing opinions, providing feedback)	Involvement	Collaboration	Empowerment
Collect information from residents/practitioners; One-way communication between residents/practitioners and the proponents of participation	Reach out to residents/practitioners to collect their opinions and feedback; One-way communication between residents/practitioners; limited to one-time consultation	Work together with residents/practitioners as contributors to ensure that concerns, aspirations, and perceptions are considered to some extent; Two-way communication between the residents/stakeholders and the proponents of participation	Work together with residents/practitioners by sharing their ideas and propositions; Higher and multiple levels of interaction between and among residents/practitioners and the proponents of participation	Enable residents/practitioners to shape their environment and lives by using their own resources (instead of being shaped by externals); High level of interaction and engagement among the residents/practitioners with the proponents of participation (i.e., externals)
Participatory formats support active data production and sharing or enable participants to actively collect data	Participatory formats support sharing of opinions and feedback on specific topics	Participatory formats support sharing of participants’ opinions (free exchange, not limited by closed questions)	Participatory formats support the interactive exchange of ideas and opinions among and between participants, thereby also commenting on the ideas and opinions of other contributors	Participatory formats support the initiation of own ideas, opinions, and designs; proponents are involved as external support

Furthermore, as the use of smartphones, the Internet, and visualization tools are spreading widely and providing new ways for communities and stakeholders to participate, this continuum of participation that advertently focuses on the decision-making power is argued to be limited because digital aspects are not adequately reflected. It is argued that digital technologies in participation provide opportunities to transcend geographic boundaries; provide instant and real-time communication and various forms of interactivity that encourage participants to actively contribute their thoughts and opinions; and enable like-minded individuals to connect, share experiences, and engage in meaningful discussions [27]. It is therefore relevant to explore the degree of involvement and the interactions between participants and proponents of plans and programs imparted by digital participatory format rather than to merely focus on the participants’ roles in the decision-making as presented in Table 1.

## Role of digital technologies for participation in a healthy urban planning context

In this section, we present selected case studies in which digital technologies have been used with residents and practitioners for varying degrees of involvement in healthy urban planning topics. We conducted a scoping review of the digital tools in the context of healthy urban planning, and we selected these cases based on our preliminary results. We chose examples that fit very well to the different categories of Table 1 and are easy to understand for the readers without a very long explanation of the study design and methods.

*Information* produced by participants about their neighborhoods, or so-called bottom-up information, can provide valuable insights to inform the planning and design of interventions that are better tailored to the community’s specific context, needs, and priorities. However, participants’ engagement levels in such projects are usually limited to collecting data. Most crowdsourcing and contributory citizen science projects fall into this category. For example, in the Citi-Sense project [28], citizens were recruited to evaluate the chosen sites for their acoustic comfort. Depending on the participants’ availability, participants observed four urban places where they used a mobile-based app to detect noise events. Once such events were detected, the app allowed sharing of the perceptions regarding the noise events, thus enabling the simultaneous collection of objective and subjective data on site. By engaging participants in actively collecting data and information on acoustics in urban spaces, the project anticipated educating about and raising awareness of the need to improve acoustic comfort in public spaces. Nevertheless, as suggested by Hasler et al. [29], the role of participants was mainly limited to sensors. Similarly, in the Digital Assessment of Subjective Environmental Exposure and Environmental Injustice (DASEIN) project conducted within the Leibniz ScienceCampus Digital Public Health [30], researchers recruited students to understand various environmental exposures that individuals face in their neighborhoods on a daily basis and at several times a day. Students were also asked to rate these exposures in terms of fairness and justice by comparing their own exposures to the exposures of others and stating whether they perceived the distribution of exposure to be equally distributed in their municipality. The participants were enabled to use an app-based questionnaire to collect their exposures in real time and several times a day for 30 days. This feasibility study concluded that the digital participatory method could be implemented in both the developed and developing country context. The study also demonstrated that amid the restrictions due to the COVID-19 pandemic, it was feasible for the researchers to provide online training and support for the participants throughout the study period.

In planning and design, participants are often asked about their opinions or feedback on plans, projects, or themes. In such cases, the engagement is usually one-way and limited to a one-time *consultation* during which participants are asked specific questions. Previously, this would translate, for example, to surveys, focus group discussions, or interviews that are time and resource intensive. With digital technologies such as online surveys and web GIS, feedback about projects can be gathered from many participants in less time. For example, the city of Cincinnati, Ohio, USA, set up a web GIS crowdsourcing tool called Shareabouts to collect residents’ opinions about the feasibility of a bike-share program [31]. Residents were invited to use the web GIS to locate desired locations for bike-share stations on a map of Cincinnati. They were able to suggest new locations for bike-share stations or support existing locations by clicking a support button. They were also asked to describe the reason for supporting or opposing locations. As a result, the tool generated 330 suggested locations and 503 comments from 206 engagements. Additionally, various locations were supported 1773 times by the participants. Of those participants who volunteered to provide personal information, 54% were male, 30% were female, and 16% did not specify. Similarly, Rayan et al. [32] used online surveys to determine and assemble sustainable urban green infrastructure (UGI) planning indicators from diverse stakeholders. The surveys allowed participants to rate the given indicators and identify other UGI indicators. A total of 212 purposively targeted stakeholders participated: decision-makers, academicians, practitioners, and students. As such, the data collected were of high quality, as the authors claimed.

During an *involvement*, the role of participants becomes more prominent in a project. Participants often have possibilities of two-way exchanges with the proponent of participation, and the method of inquiry is not limited to closed questionnaires. Nonetheless, it is limited to specific phases of planning. For example, in the study by Barrie et al. [33], elderly citizens were involved in order to explore how and why they engage with public green spaces. The elderly citizens not only collected data with the assistance of the researchers but were also later engaged in the preliminary analysis of the data and contributed feedback and ideas on the methods, process, audit tools, and design of the larger project. In this case, digital technology in the form of a smartphone-based app was used as a tool for the participants to record their experiences and perceptions of using public spaces and to collect geolocations, photographs, and additional comments. Together with the researchers, the participants discussed how the collected data could or should be analyzed and critically looked at their neighborhoods and living environments to reflect on and understand various aspects of their neighborhoods.

**Fig. 1:**
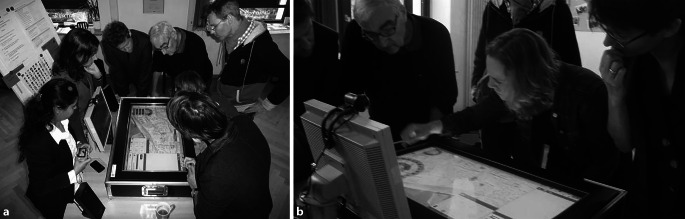
Stakeholders working around the MapTable. (Source: Shrestha et al. [34])

Further in the participation continuum is *collaboration*, at which participants are enabled to establish two-way interactions with the proponent of the participatory activity and interact with each other to share their opinions. As suggested by Hasler et al. [29], the role of participants can be seen as stakeholders rather than merely participants. Here, digital technologies could play the role of facilitators of such interactions. For example, Shrestha et al. [34] reported on how a specific digital technology using a horizontal table surface with a touch sensitive screen, the MapTable-based digital tool (Fig. 1), could be used to support the exchange of knowledge and opinions among stakeholders from various sectors such as urban planning, health departments, and social planning on issues that were affecting the health of people in Dortmund, Germany. By using the digital tool, participants could view different environmental and social indicators interactively, be immersed in evidence-based discussions with each other, add their knowledge through sketch mapping, and produce a joint picture of issues affecting people’s health. The researchers reported the benefits of integrating the rich picture method as a nondigital tool to expand the opportunity for participants to address broad issues affecting health situations. The research also highlighted the critical role of a facilitator to ensure equal opportunity for participants to voice their opinions, as well as to provide tool-related support. In another example, Baba et al. [35] showed how an online deliberative platform could be used as a debate forum for community members to clarify the perceptions and attitudes toward climate change adaptation in disaster prevention. An online debate forum was created where participants discussed and deliberated on the topic. The forum was positively evaluated for its potential use for any geographic region, by which it provided more opportunities to interact at different times. The forum elicited many responses and ideas from participants because it was made available over a relatively long period. This enabled the participants to conduct their own research before and during online deliberation. However, compared to face-to-face deliberation, online deliberations are relatively unstructured without a facilitator and less constrained in building consensus and subsequent action, which leads to less satisfaction among the participants in prioritizing policy solutions. Because discussions often centered around a few individuals who expressed their views frequently, participants also raised concerns about the risk of reinforcing inequalities and creating negative emotions.

For projects aimed at *empowering* participants, digital tools are often used to collect data on the local environment. However, in contrast to lower-level engagement, the participants use the data collected to produce action-oriented results. For example, in the Our Voice project [36], elderly citizens were engaged to identify barriers and facilitators of walking in their neighborhood and to propose improvement actions. The participants used a mobile app called Discovery Tool to document local environmental features through geocoded photographs and audio narratives in their walking routes. They further analyzed these data to propose actions for improvement during face-to-face workshops and presented these proposals to the members of the municipal council. In another project by Adelle et al. [37], residents used electronic tablets to create a digital story of their personal experience of food environments in their neighborhood. The participants were empowered to voice their concerns to the authorities as they later presented the collected data alongside scientific data from researchers in the Food Governance Community of Practice.

## Discussion and conclusions

Digital technology can support participation by enabling different levels of engagement. With the examples presented above, we can ascertain that digital technology can be a means to enable both one-way and two-way interactions. These interactions span from residents/practitioners sharing data and providing opinions or feedback on specific topics to residents being empowered to develop solutions and demand actions from relevant authorities.

The examples presented above show that some forms of engagement can be supported entirely within digital platforms, in particular when engaging citizens to collect data about environmental factors. Those projects in which residents are engaged as sensors to collect objective and subjective data on environmental situations are found to be well supported with digital platforms. Similarly, digital technology such as online platforms and surveys provide opportunities to reach more people and stakeholders with diverse profiles than on-site meetings can, while collecting feedback on already designed plans or programs. However, it is often argued that data produced online are seldom representative [29]. This situation arises especially when the online platform is designed to allow participation without registration and without sharing basic information, as indicated in the case of Cincinnati [31]. Nonetheless, targeted online consultation can also be designed. This was shown in the case of the UGI [32], where purposeful sampling resulted in both the desired representativeness and outreach of participants.

In the case of higher engagement, the presented examples suggest that other nondigital formats and traditional methods, such as follow-up workshops and focus group discussions, are necessary to complement the digital form of participation in order to get deeper insights into the problems and to codevelop solutions. As presented, participants can collaborate effectively to understand each other’s opinions and priorities. Digital platforms have made it possible for diverse residents to deliberate the interest of the actual community in a secure space and to know the opinions of others in the community. Understanding each other’s opinions may therefore not always require face-to-face interaction. However, physical face-to-face interaction can be valuable in some situations, especially when the group needs to reach a consensus or prioritize policy codesign solutions, as in the example of online deliberation. Having face-to-face interaction allows the exchange of nonverbal cues that might convey important information as well as nuances that are necessary to build trust. Opportunities for instant feedback and clarification during face-to-face interactions could therefore lead to a quicker resolution of issues.

Complex multisectoral problems such as environmental health inequalities require a deep level of understanding and exploration from various sectors, and a platform that combines digital technologies with face-to-face participatory analysis may help to fully capitalize on the innovativeness of digital technologies, as presented in the case of using interactive MapTable [34]. Moreover, supplementing quantitative data with qualitative data introduces context, meaningful perspectives, and multiple viewpoints. As suggested by Knigge and Cope [38], such supplementation will better reflect the complexities of the social world over time and space. In this regard, digital technologies could be used innovatively with physical face-to-face methods of participation to include narratives sourced directly from affected individuals, thereby offering an opportunity for democratization of knowledge, which ultimately produces more holistic and sociopolitically relevant takeaways. This was shown in the Our Voice project and the digital storytelling project.

Amid the risk of low digital literacy as one of the causes of the digital divide and with elderly people possibly being digitally excluded [39], we can agree that the young generation requires less training on using technology. Digital technology may thus create immense opportunities for the young generation to be involved in the active generation of data for planning. Nevertheless, studies requiring deeper exploration and codevelopment of solutions with elderly citizens can also take advantage of digital innovativeness if such studies are carefully designed, can reduce the technical burdens on the elderly, and can give more guidance.

Finally, from the presented case studies, we can find diverse possible uses of digital technology to inform health-oriented planning. While some of these tools and data produced via the tools are also being used in actual planning processes (e.g., Cincinnati and Our Voice), several tools are often used for case studies and are seldom employed in actual planning practices. Since opportunities for participation are often strongly linked to planning practices, it is nonetheless essential for the proponents of the participation activity to know what information is needed and how to choose from a plethora of digital tools in order to use the right one.

## Declarations

For this article no studies with human participants or animals were performed by any of the authors.
